# Therapeutic Potential of Sitafloxacin as a New Drug Candidate for *Helicobacter* Eradication in Korea: An In Vitro Culture-Based Study

**DOI:** 10.3390/antibiotics10101242

**Published:** 2021-10-13

**Authors:** Youn I Choi, Sung Min Lee, Jun-Won Chung, Kyoung Oh Kim, Kwang An Kwon, Yoon Jae Kim, Jung Ho Kim, Sun Mi Lee, Jin-Yong Jeong, Dong Kyun Park

**Affiliations:** 1 Department of Gastroenterology, Gil Medical Center, Gachon University, Incheon 21565, Korea; cys7like@hanmail.net (Y.I.C.); yismin77@naver.com (S.M.L.); kkoimge@naver.com (K.O.K.); toptom@gilhospital.com (K.A.K.); yoonmed@gachon.ac.kr (Y.J.K.); junghokimm@gilhospital.com (J.H.K.); pdk66@gilhospital.com (D.K.P.); 2 Asan Medical Center, Asan Institute for Life Sciences, University of Ulsan College of Medicine, Seoul 05505, Korea; eclipse-lsm@hanmail.net (S.M.L.); jyjeong@amc.seoul.kr (J.-Y.J.)

**Keywords:** sitafloxacin, quinolone series, *Helicobacter pylori*, eradication

## Abstract

Background: Increased prevalence of antibiotic resistance to *Helicobacter pylori* (*H. pylori*) infection worldwide has driven the search for a new therapeutic candidate. Recently, sitafloxacin, a novel 4-quinolone agent, has emerged as a new therapeutic option for *H. pylori* eradication, in Japan. However, data on its efficacy for *H. pylori* eradication in Korea are limited. Therefore, we aimed to investigate the therapeutic potential of sitafloxacin as a first-line treatment for patients with *Helicobacter* infection through gastric tissue culture-based studies. Materials and Methods: We prospectively enrolled treatment-naïve patients with *H. pylori* infection who visited the Gil Medical Center between March 2015 and March 2018. After obtaining written informed consent from patients, a total of 121 *H. pylori* strains were collected. We tested the susceptibility of these strains to sitafloxacin, and other antibiotics for Helicobacter eradication, including clarithromycin (CLR), metronidazole (MTZ), amoxicillin (AMX), tetracycline (TET), levofloxacin (LEV), and ciprofloxacin (CIP) using the agar dilution technique. The minimum inhibitory concentration (MIC) of these antibiotics against *H. pylori* strains were determined. Results: None of the *H. pylori* strains obtained were resistant to sitafloxacin (MIC > 1, *n* = 0), while other conventional eradication drugs including CLR, MTZ, AMX, and TET showed 24.8% (*n* = 30), 30.6% (*n* = 37), 5.0% (*n* = 6), and 0.8% (*n* = 1) resistance, respectively. Compared to the resistance rates of other quinolones (LEV [36.4%, *n* = 44] and CIP [37.2%, *n* = 45]), sitafloxacin showed the best antibiotic performance against Helicobacter strains (0%, *n* = 0). Furthermore, sitafloxacin also inhibited the growth of 14 *H. pylori* strains (12.4%), which were resistant to both of clarithromycin, and metronidazole, and 27 strains (22.3%) with multidrug resistance. Conclusions: Sitafloxacin might be a new promising candidate for Helicobacter eradication where antibiotic resistance for Helicobacter is an emerging medical burden, such as in Korea.

## 1. Introduction

The antibiotic resistance associated with *Helicobacter pylori* *(H. pylori)* infections has increased worldwide, leading to a global medical burden [1,2]. Concerns regarding multi-drug resistant (MDR) *Helicobacter* infections have been increasing [3]. Recent reviews regarding the status of MDR *H. pylori* worldwide showed that, even though it varied among and within nations, the pooled prevalence of *H. pylori* primary resistance to clarithromycin and metronidazole was up to 32% and 38%, respectively, in Europe [3,4,5], and 27%, and 59%, respectively, in Southeast Asia [3,5]. Fluoroquinolone, a component of the second-line or rescue regimen for drug-resistant *H. pylori*, has been gaining resistance to *H. pylori* infection according to a 10-year trend analysis, from a 12% prevalence in 2006–2008 to 31% in 2021–2016. In 2016, considering public health and social situations worldwide, WHO showed the priority list of antibiotic-resistant bacteria which should be overcome through global strategy; drug-resistant *H. pylori* was not only included on the priority list, but was also designated a high-priority bacteria [6].

Also in Korea, increased pattern of antibiotic resistances of *H. pylori* has been emerging issues [7,8]. According to a recent report on the antibiotics resistance crisis against *H. pylori* in Korea, resistance rates of amoxicillin and clarithromycin were approximately 20~25% and 25~30%, respectively [7]. Metronidazole, tetracycline, and levofloxacin, which are generally used as secondary or rescue regimen for *H. pylori,* showed resistance rates of 30%, 20%, and 45%, respectively [7]. Another report from Korea also investigated the primary and secondary resistance rate against *H. pylori* over the past decades, and showed that primary resistance rate of clarithromycin, and metronidazole, and levofloxacin against *H. pylori* increased over time [8]. 

Several factors linked to the emergence of MDR *H. pylori* have been identified [4,9]: first, antibiotic misuse and overuse by patients, including over-prescription by physicians along with increased national antibiotic consumption; second, treatment failure on account of patient factors, including poor compliance, improper indication, and so on; and third, treatment failure due to bacterial factors, including varying mutations, efflux pumps, biofilm formation, and so on [4,9]. 

To overcome the aforementioned issues associated with the increased prevalence of MDR *H. pylori* infections, national campaigns for proper antibiotic use, countermeasures for patient-oriented treatment failure factors, and antibiotic sensitivity tests for detecting drug-resistant *H. pylori* have been increasing in clinical practice, highlighting the need for the discovery of new therapeutic drugs for *H. pylori* infection. 

Recently, sitafloxacin, a novel 4-quinolone agent with bactericidal effects, emerged as a new therapeutic option for *Helicobacter* eradication [10,11,12]. Recent reports on the most common type of MDR *H. pylori* infection have pointed to the triple resistance to fluoroquinolones, metronidazole, and clarithromycin, so new quinolones might counteract MDR *H. pylori* [4]. 

However, data on the efficacy of sitafloxacin for *Helicobacter* eradication outside Japan are limited. In Korea, although sitafloxacin is used as an antibiotic, it has not been used as a *Helicobacter* eradication regimen. In Japan, even though there have been several studies investigating the therapeutic potential of sitafloxacin in *H. pylori* infections, the antibiotic sensitivity of *H. pylori* varies among nations, owing to different levels of antibiotic use [3,9,13,14]. *H. pylori* infection status and sitafloxacin efficacy should be validated outside Japan to determine if indeed sitafloxacin can be used as a treatment for *H. pylori* infection.

Therefore, we aimed to investigate the therapeutic potential of sitafloxacin as a first-line treatment for patients with *Helicobacter* infection through in vitro and gastric tissue culture-based studies.

## 2. Results

### 2.1. Clinical Characteristics of the Enrolled Study Population

The baseline characteristics of the enrolled population were as follows. The mean age of patients was 54.6 ± 12.5 years, and 50.4% (*n* = 61) were male (Table 1). The most common cause of eradication was peptic ulcer disease (67.8%; *n* = 82) (Table 1).

### 2.2. Results for Antimicrobial Susceptibility Testing (Sitafloxacin vs. Other Conventional Drugs)

Of the 121 *H. pylori* strains, none were resistant to sitafloxacin (MIC > 1, *n* = 0), whereas other conventional drugs used for eradication, including CLR, MTZ, AMX, and TET, showed resistance rates of 24.8% (*n* = 30), 30.6% (*n* = 37), 5.0% (*n* = 6), and 0.8% (*n* = 1), respectively (Table 2). Other conventional quinolones, such as LEV and CIP, showed 36.4% (*n* = 44) and 37.2% (*n* = 45) resistance, respectively (Table 2).

**Figure 1:**
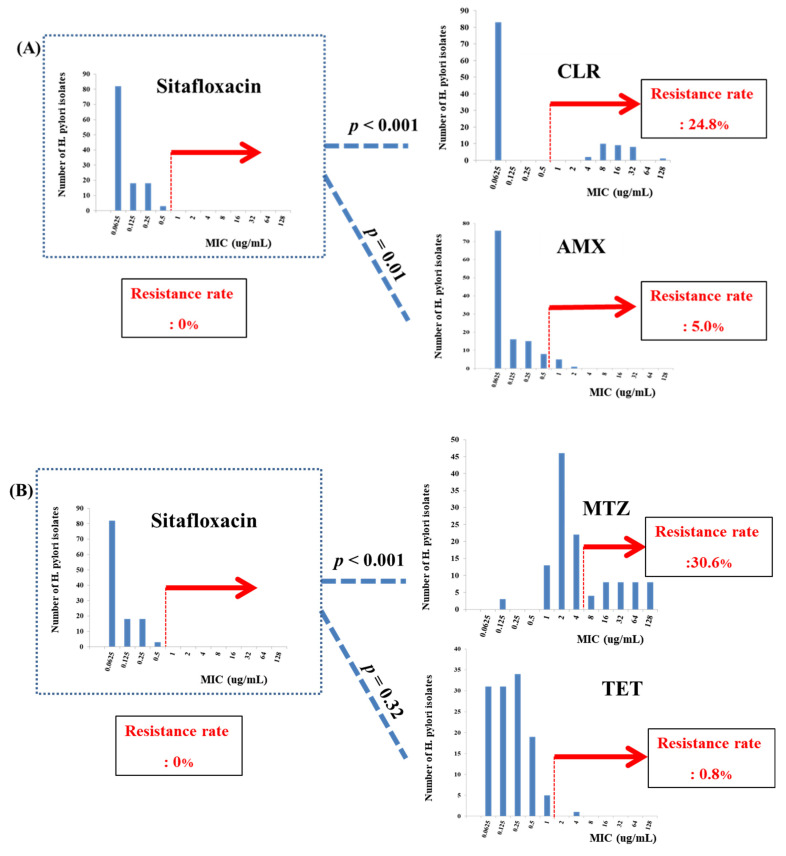

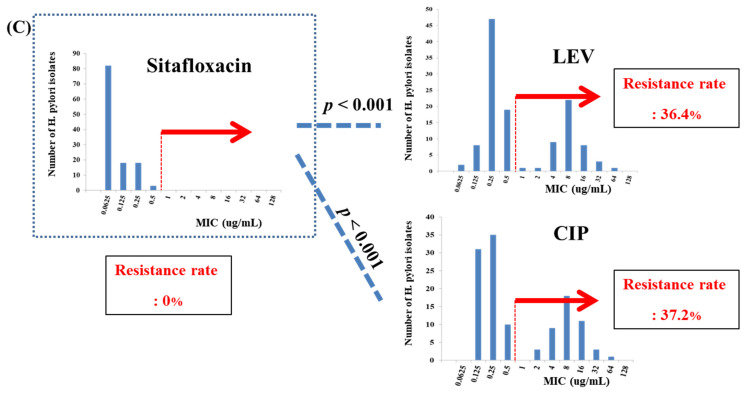
Antibiotic activity comparisons among sitafloxacin and other drugs. (**A**) Sitafloxacin, (**B**) Levofloxacin, and (**C**) Ciprofloxacin.

When we compared sitafloxacin activity with that of CLR, MTZ, and AMX, sitafloxacin showed significantly excellent eradication activity against these antibiotics (*p* < 0.001, *p* < 0.001, and *p* = 0.01, respectively) (Figure 1A,B). However, there was no statistically significant difference between the antimicrobial activity of sitafloxacin and TET (*p* = 0.32) (Figure 1B). 

### 2.3. Results for Antimicrobial Susceptibility Testing (Sitafloxacin vs. Other Quinolone Drugs)

Compared to the resistance rates of other quinolone drugs (LEV [36.4%, *n* = 44] and CIP [37.2%, *n* = 45]), sitafloxacin showed the best antibiotic performance against *Helicobacter* strains (0%, *n* = 0), with statistical significance (*p* < 0.001) (Figure 1C).

### 2.4. Results of Antimicrobial Susceptibility Testing (Sitafloxacin on MDR Helicobacter)

Furthermore, sitafloxacin also inhibited the growth of 14 strains (12.4%), which were co-resistant to CLR and MTZ, and 27 strains (22.3%) with multidrug resistance activity (resistance to >2 drugs).

## 3. Discussion

In this study, we investigated the in vitro activity of sitafloxacin, a new-generation quinolone drug, in the treatment of *H. pylori* infection through gastric tissue culture-based studies using *H. pylori* strains obtained from 121 patients with *H. pylori* infection in Korea. According to our study results, sitafloxacin showed a 0% antibiotic resistance rate for *H. pylori*, and successfully acted against all 14 strains of multidrug-resistant *H. pylori* (resistant to ≥2 drugs). To overcome bacterial factor-oriented treatment failure, along with antibiotic sensitivity tests, new therapeutic targets and other novel drugs can be used as part of the *Helicobacter* eradication strategy. The results of this study showed the possibility of sitafloxacin as a new drug candidate for regimens in *H. pylori* infection in Korea, where fluoroquinolone resistance has increased over the years.

Sitafloxacin, a novel fourth generation quinolone, effectively acts against a broad spectrum of gram-negative and gram-positive bacteria (including anaerobes), as well as atypical pathogens [12]. Sitafloxacin was discovered in Japan [12,15] and was first used in *Helicobacter* eradication as a rescue regimen or as an alternative first-line treatment for patients with penicillin allergies [10,11,16,17,18,19,20,21,22]. The background of the need for sitafloxacin in *H. pylori* eradication practice is as follows. Conventional quinolone series drugs, especially levofloxacin and moxifloxacin, act as bacteriostatic agents against *H. pylori* strains, and are components of a second-line or third-line rescue therapy [23,24]. However, along with other conventional anti-*Helicobacter* drugs such as clarithromycin and metronidazole, quinolone series drugs have also shown increased antibiotic resistance annually due to increased consumption of these drugs in respiratory tract infections, urinary tract infections, and other infections other than *H. pylori* infections [25,26]. These trends were observed to be limited in Korea or Japan, but other nations also struggle with conventional quinolone resistance [5,26,27,28,29]. According to a recent report, the resistance of *H. pylori* to clarithromycin, metronidazole, and levofloxacin has reached alarming levels of up to 46%, even though it varied among countries [5]. Referring to previous research on the antibiotic resistance status in Korea, the primary resistance rate for *H. pylori* infection significantly increased from 4.5% in 2003 to 62.2% in 2018 [8].

Sitafloxacin is not yet approved for global use; however, it was recently used in Japan and Thailand. Even though sitafloxacin has been used in Japan as an anti-*Helicobacter* medication, there is limited data on its efficacy in *Helicobacter* eradication elsewhere, including in Korea. We searched the PubMed platform to find research articles (Language: English, original article, keywords: *Helicobacter* AND sitafloxacin) between January 2010 and January 2021 and found only two articles published outside Japan. First, Miftahussurur et al. reported the in vitro activity of sitafloxacin in 98 *H. pylori* strains from Nepal and Bangladesh after performing a whole-genome mutation analysis. According to the study results, 95.2% of *H. pylori* strains from Nepal and 98.2% of strains from Bangladesh showed high susceptibility to sitafloxacin. Korea, Nepal, and Bangladesh are classified as regions with a high prevalence of *H. pylori* infection (Bangladesh at 60.2% prevalence), and high antibiotic resistance, including that of clarithromycin, metronidazole, and levofloxacin (39.3%, 94.6%, and 66.1%, respectively), resulting in eradication failure [30]. Second, Miftahussurur et al. reported the possibility of sitafloxacin in *H. pylori* infection using 63 *H. pylori* strains from Dominican patients [31]. In the Dominican Republic, physicians struggled with the high prevalence of *H. pylori* resistance to levofloxacin and metronidazole. Similar to our study results, they also revealed that all collected strains were susceptible to sitafloxacin. Despite the limited number of studies, three studies performed outside Japan, including our current research, reported that sitafloxacin showed excellent in vitro antibiotic activity against *H. pylori* infection.

In Japan, based on the stacked in vitro activity data of sitafloxacin from Sanchez et al. [32], several clinical studies have been conducted in Japan using sitafloxacin as a *Helicobacter* eradication regimen, including primary or rescue therapy. Sitafloxacin-based triple regimens usually consist of sitafloxacin (100 mg bid)/proton pump inhibitor (PPI) along with MTX (250 mg bid) or AMX (750 mg bid or 500 mg qid or 250 mg bid). With a variety of combinations for sitafloxacin-based triple regimens, several researchers investigated its efficacy in *Helicobacter* eradication as compared to other conventional regimens as first line or rescue regimen. To verify sitafloxacin as the first line regimen, Sugimoto et al. (study period: 2011–2015, Japan) investigated the efficacy of sitafloxacin containing a triple regimen (sitafloxacin 100 mg bid + PPI + MTX 250 mg bid for 1 week) among 45 treatment naïve *H. pylori* infection patients, and they showed eradication success rate (ESR) of 100% in intention-to-treat analysis (ITT), and 92.7% in per protocol (PP) analysis [21]. Matsuzaki et al. (study period: 2009–2011, Japan) investigated the efficacy of sitafloxacin-based rescue therapy (sitafloxacin 100 mg bid + PPI + AMX 500 mg qid for 1 week) among first- and second-line eradication failure patients, and they showed 78.2%, and 83.6% of ESR in ITT, and PP analysis, respectively [19]. Murakami et al. (study period: 2009–2011, Japan) investigated the efficacy of sitafloxacin-based therapy (sitafloxacin 100 mg bid + PPI + AMX 750 mg qid for 1 week) as compared to LVF-based therapy (levofloxacin 300 mg bid + PPI + AMX 750 mg qid for 1 week) to establish a third-line eradication as an multicenter, randomized controlled trial [33]. According to this study, the sitafloxacin-based therapy group showed statistically higher ESR of 70%, and 72.1% in ITT, and PP analysis, as compared to LVF-based therapy (ESR of 43.1%, and 43.7% in ITT, and PP analysis, respectively) [33]. Mori et al. conducted a study to determine the 10-year trend of the efficacy of a sitafloxacin-based triple therapy as a third-line regimen, and found that its efficacy did not change from 2009 to 2015, with eradication success rates over 80% [34]. Even more, sitafloxacin has also shown efficacy against levofloxacin-resistant strains. In Korea, levofloxacin-resistant *H. pylori* infection is an emerging issue. Therefore, with these data, further randomized controlled studies to validate the efficacy of sitafloxacin as a rescue regimen in Korea should be conducted.

As mentioned above, even in Japan, several studies have investigated the therapeutic potential of sitafloxacin in *Helicobacter* infection, which varies among nations for antibiotic sensitivity of *H. pylori* infection owing to different consumption of national antibiotic use, and *H. pylori* infection prevalence status; however, its efficacy should be validated outside Japan in *H. pylori* infection treatment. Further clinical studies using sitafloxacin outside Japan should be performed. Our study results pertaining to the in vitro activity of sitafloxacin against Korean *H. pylori* strains might serve as a basis for further clinical research in Korea.

The possible underlying mechanisms of action of sitafloxacin against drug-resistant *H. pylori* have been previously determined [10,17,18,34]. Sitafloxacin has been found to successfully act against *H. pylori* with mutations in the *gyrA* gene, one of the hotspots of the quinolone resistance-determining gene [34]. The *gyrA* genes of *H. pylori* strains encode DNA gyrase, an essential enzyme that maintains the helical structure of DNA, and is involved in DNA replication, recombination, and transcription [35]. Conventional quinolones bind to and inhibit DNA gyrase, resulting in irreversible DNA damage, which then acts as a bacteriostatic agent [35]. However, drug-resistant *H. pylori* strains have evolved to evade attachment from conventional quinolone series drugs though mutations in their *gyrA* gene [35]. Therefore, since the *gyrA* gene plays a pivotal role in nucleic acid synthesis, mutations in this gene may result in the resistance of *H. pylori* to conventional quinolone drugs [10,22,36,37]. Notably, among the various sites of *gyrA* gene mutations, sitafloxacin especially showed activity at D91, but not at N87. If patients infected with *H. pylori* with *gyrA* mutation at N87 were diagnosed, a third-line regimen other than sitafloxacin should be prescribed [34].

There are several limitations to this study. First, we enrolled patients with *H. pylori* infections who visited the Gil Medical Center, which is located in Incheon, Korea. Since the antibiotic resistance status of *H. pylori* differs among geographic regions and time, caution should be exercised when applying our results to other locations. Second, even though we included *H. pylori* strains from 121 patients and enrolled a relatively large number of strains to draw conclusions compared to those of previous studies, further multicenter studies are needed. Third, because antibiotic exposure history was dependent on patients’ recall, natural bias from recall might have affected our study results. Fourth, since our study aimed to investigate the antibiotic resistance status of *H. pylori* for sitafloxacin through an in vitro stomach tissue culture-based assay and results from antibiotic sensitivity tests, real-world clinical practice might differ, so further clinical studies using sitafloxacin as a *Helicobacter* eradication regimen for patients with *H. pylori* infection should be further considered. Our study results may then be the basis for further clinical research on this. Fifth, in this study, we focused on whether or not sitafloxacin, a novel antibiotic for the Korean general population, showed potential activities against *H. pylori* infection. In this regard, we did not further investigate the acquired double mutations in the *gyrA* gene at the A87 and D91 positions on the *H. pylori* species owing to time and cost issues. Further investigation should be guaranteed. Sixth, *H. pylori* can acquire double mutations in the *gyrA* gene at the A87 and D91 positions, which results in drug resistance against sitafloxacin [30]. Furthermore, because *H. pylori* also has traits of cross-resistance with other quinolone series drugs such as ciprofloxacin, careful clinical antibiotic use of sitafloxacin for appropriate indications, as well as the use of antibiotic sensitivity test–based regimens rather than empirical use, are still imperative to reduce the possibility of antibiotic resistance. Further studies should be guaranteed to investigate the efficacy of sitafloxacin in the presence of double mutations in the *gyrA* gene at the A87 and D91 positions of *H. pylori* strains.

Despite the aforementioned limitations, our data on the efficacy of sitafloxacin against *H. pylori* eradication showed that the in vitro efficacy of sitafloxacin was proven in *Helicobacter pylori* infection outside Japan, in Korean patients. Additional clinical studies regarding sitafloxacin against *H. pylori* infection should be conducted.

## 4. Materials and Methods

### 4.1. Institutional Review Board Approval

This study was conducted in accordance with the Declaration of Helsinki, and the study protocol was approved by the ethics committee of the Gil Medical Center. The Institutional Review Board of Gil Medical Center reviewed the study protocol and ethics (GAIRB2016-329).

### 4.2. Enrollment of Patients with H. pylori Infections

We prospectively enrolled patients in the Gil Medical Center with *Helicobacter* infection, no previous history of *Helicobacter* eradication, and no history of antibiotic use within 2 years between June 2016 and March 2019. After obtaining written informed consent from patients who agreed to obtain stomach biopsy and tissue cultures for antibiotic sensitivity testing, a total of 121 *H. pylori* strains from all patients were collected.

### 4.3. Isolation and Cultivation of H. pylori Strains from Gastric Tissue Samples

The isolation of *H. pylori* from the stomach biopsy samples of 121 patients was performed as follows, the detailed process of which was illustrated in a previous publication by our group [38]. Immediately after the stomach tissue was obtained, the specimens were kept in a transport medium and delivered to the laboratory for analysis. Performing basic aseptic techniques throughout the process, stomach biopsy specimens were supplemented with 5% sheep blood containing vancomycin (10 μg/mL), amphotericin B (5 μg/mL), trimethoprim (5 μg/mL), and polymyxin B (2.5 IU) after crushing specimens using an aseptic surgical knife. The supplements were then cultivated under micro-ventilation conditions (5% O_2_, 10% CO_2_, 85% N_2_) at 37 °C.

We used gram staining and biochemical methods to confirm whether the bacterial colonies from the cultures were indeed *H. pylori*. To obtain *H. pylori* colonies, each *H. pylori* strain was stored in Brucella liquid medium (Difco Laboratories, Detroit, MI, USA) containing 15% glycerol at −70 °C.

### 4.4. Antimicrobial Susceptibility Testing

We further tested the antibiotic susceptibility of the *H. pylori* strains using agar dilution methods with Mueller–Hinton agar (Difco Laboratories, Detroit, MI, USA) supplemented with 5% sheep blood in accordance with guidelines from the Clinical and Laboratory Standards Institute and recent review articles [5,8,14]. The antibiotics included in this study were sitafloxacin and other conventional antibiotics for *H. pylori* eradication, including the quinolone series (clarithromycin, levofloxacin, and ciprofloxacin), amoxicillin, tetracycline, rifabutin, and furazolidone, and the broth microdilution technique was used. Each antibiotic was diluted in a medium supplemented with 5% sheep blood (obtained within 2 weeks of birth, and cooled to 80 °C) (Comed, Seoul, Korea).

Afterwards, 1 × 10^7^ colony-forming units of *H. pylori* strains cultured in blood culture medium for 72 h were inoculated on Mueller–Hinton agar containing each targeted antibiotic. After cultivation and incubation of the cultures under micro-ventilation conditions (5% O_2_, 10% CO_2_, 85% N_2_) at 37 °C for 3 days, we observed the presence of bacterial colonies. Each experiment was performed in triplicate and repeated at least three times per strain.

### 4.5. Definition of Antimicrobial Resistance: MIC Criteria

The minimum inhibitory concentration (MIC) was defined as the lowest concentration in which antibiotics completely inhibited the visible growth of *H. pylori* strains in the agar dilution test using the broth microdilution technique. The cut-off values for *H. pylori* resistance against each targeted antibiotic were as follows: sitafloxacin (>1 μg/mL), clarithromycin (>1 μg/mL), amoxicillin (>0.5 μg/mL), tetracycline (>4 μg/mL), and other quinolone series, including levofloxacin (>1 μg/mL) and ciprofloxacin (>1 μg/mL).

## 5. Conclusions

In conclusion, we showed the therapeutic potential of the novel fluoroquinolone drug sitafloxacin in the treatment of *H. pylori* infections, especially among Korean patients. Our study results may serve as a basis for further multicenter, randomized controlled clinical studies.

## Figures and Tables

**Table 1:** Demographic features of enrolled patients infected with *H. pylori* (*n* = 121).

Demographics	*n* (%)
Age, mean ± SD (years)	54.6 ± 12.5
Men, *n* (%)	61 (50.4%)
Current smoking, *n* (%)	23 (19.3%)
Current drinking, *n* (%)	46 (38.7%)
Comorbidity	
Diabetes mellitus type 2	7 (6.4%)
Hypertension	30 (25.8%)
Dyslipidemia	4 (3.2%)
Chronic liver disease	4 (3.2%)
Cerebrovascular disorders	
Reason for endoscopy, and eradication for *H. pylori*	
Peptic ulcer disease	82 (67.8%)
Early gastric cancer	14 (11.6%)
MALToma	4 (3.3%)
Atrophic gastritis	21 (17.3%)

Abbreviation: *H. pylori—Helicobacter pylori*; SD—standard deviation; *n*—number; MALToma—mucosa associated lymphoid tissue lymphoma.

**Table 2:** Prevalence of antibiotic resistance in *H. pylori* isolates.

	Resistant Breakpoint of MIC (μg/mL)	No. of Resistant Strains /Total Strains	Resistance Rate (%)
CLR	>1	30/121	24.8%
MTZ	≥8	37/121	30.6%
AMOX	≥1	6/121	5.0%
TET	≥2	1/121	0.8%
LEV	>1	44/121	36.4%
CIP	>1	45/121	37.2%
Sitafloxacin	>1	0/121	0%

Abbreviation: CLR—clarithromycin; MEZ—metronidazole; AMX—amoxicillin; TET—tetracycline; LVX—levofloxacin; CIP—ciprofloxacin; No.—number; *H. pylori*—*Helicobacter pylori*; MIC—minimum inhibitory concentration.
